# Tigers Need Cover: Multi-Scale Occupancy Study of the Big Cat in Sumatran Forest and Plantation Landscapes

**DOI:** 10.1371/journal.pone.0030859

**Published:** 2012-01-23

**Authors:** Sunarto Sunarto, Marcella J. Kelly, Karmila Parakkasi, Sybille Klenzendorf, Eka Septayuda, Harry Kurniawan

**Affiliations:** 1 Department of Fish and Wildlife Conservation, Virginia Tech, Blacksburg, Virginia, United States of America; 2 Species Program, Worldwide Fund for Nature-Indonesia, Jakarta, Daerah Khusus Ibukota, Indonesia; 3 Species Program, World Wildlife Fund, Washington, D.C., United States of America; Smithsonian's National Zoological Park, United States of America

## Abstract

The critically endangered Sumatran tiger (*Panthera tigris sumatrae* Pocock, 1929) is generally known as a forest-dependent animal. With large-scale conversion of forests into plantations, however, it is crucial for restoration efforts to understand to what extent tigers use modified habitats. We investigated tiger-habitat relationships at 2 spatial scales: occupancy across the landscape and habitat use within the home range. Across major landcover types in central Sumatra, we conducted systematic detection, non-detection sign surveys in 47, 17×17 km grid cells. Within each cell, we surveyed 40, 1-km transects and recorded tiger detections and habitat variables in 100 m segments totaling 1,857 km surveyed. We found that tigers strongly preferred forest and used plantations of acacia and oilpalm, far less than their availability. Tiger probability of occupancy covaried positively and strongly with altitude, positively with forest area, and negatively with distance-to-forest centroids. At the fine scale, probability of habitat use by tigers across landcover types covaried positively and strongly with understory cover and altitude, and negatively and strongly with human settlement. Within forest areas, tigers strongly preferred sites that are farther from water bodies, higher in altitude, farther from edge, and closer to centroid of large forest block; and strongly preferred sites with thicker understory cover, lower level of disturbance, higher altitude, and steeper slope. These results indicate that to thrive, tigers depend on the existence of large contiguous forest blocks, and that with adjustments in plantation management, tigers could use mosaics of plantations (as additional roaming zones), riparian forests (as corridors) and smaller forest patches (as stepping stones), potentially maintaining a metapopulation structure in fragmented landscapes. This study highlights the importance of a multi-spatial scale analysis and provides crucial information relevant to restoring tigers and other wildlife in forest and plantation landscapes through improvement in habitat extent, quality, and connectivity.

## Introduction

Although tigers (*Panthera tigris* Linnaeus, 1758) globally inhabit a variety of habitat types and are able to adapt to a wide range of environmental conditions [1], in Sumatra they are generally believed to live only in natural forest areas. Habitat loss has widely been recognized as the main threat to Sumatran tigers [2]. Forest conversion, therefore, has typically been equated to tiger extermination. In Sumatra, natural forests have largely been converted to forestry and agricultural plantations. Information from local people and our preliminary surveys indicate, however, that such plantation areas are not totally useless for tigers. With recent and future changes in Sumatra landscapes and across the tiger range involving continued conversion of forests into plantations, it is crucial to understand whether existing plantation areas are useable by tigers. Furthermore, for tiger restoration, it is also important to understand how habitat conditions within forests and plantations can be improved.

The use of habitats by Sumatran tigers within, and especially outside of, natural forests has barely been studied. Previous studies have largely focused on population estimation in intact forests and/or within protected areas [3], [4], [5]. Only recently have some investigators begun assessing the value of non-pristine forests as tiger habitat [6]. Except for Maddox et al. [7], who investigated tigers in a non-cultivated conservation area within an oilpalm concession, there is no other study conducted in Sumatra examining use of non-forest areas. This study is the first that systematically investigates occupancy and habitat use by Sumatran tigers in different landcover types within a multi-use landscape. We focused on Riau Province in central Sumatra, which historically was considered by Borner [8] as the stronghold for Sumatran tiger conservation.

### Distribution and habitat models

Knowledge of distribution and habitat requirements of animals are key elements in ecology and basic prerequisites for effective wildlife management [9], [10]. It also is important to construct reliable predictive models of animal occurrence based on solid understanding of the relationships between animals and habitat. Such models are urgently needed for wildlife management, but constructing them for rare, elusive, and highly mobile species such as the Sumatran tiger is a demanding task. Due to data limitations, the distribution of tigers is often broadly mapped based on historical records in combination with general knowledge and expert opinion regarding perceived potential habitats.

Understanding patterns of animal distribution requires consideration of the scale appropriate to address wildlife conservation needs [9], [11], [12] because habitat selection, one of the determining factors in animal distribution, takes place at a variety of spatial and temporal scales [13], [14], [15]. While broad-scale tiger distribution maps such as the Tiger Conservation Unit [16] or the updated version, Tiger Conservation Landscapes [17], have been useful to direct conservation strategies at the global level, they are limited when it comes to local or regional landscape-level management. Therefore, distribution models should consider appropriate scale (spatial and temporal), predictive ability, and include an assessment of uncertainty.

In this study, we use an occupancy modeling approach [18], [19], [20], [21] that incorporates the probability of detection into the estimation procedure, recognizing that it is not always 1.0 [22]. This provides a more accurate depiction of animal distribution without the need to assume that all animals present in the surveyed area are detected [21]. Application of such a technique has been done for some groups of animals including tigers in Kerinci-Seblat [23] and the large mammal community in India [24]. By incorporating covariates into the models it is possible to describe the geographic range and habitat characteristics of the species of interest in the surveyed area [25], [26], and also predict the probability of occurrence for other sites not surveyed.

### Multi-scale analysis

Recognizing that multiple-scale processes affect tiger distribution [27], we developed models depicting tiger-habitat relationships at multiple scales. In addition to estimating tiger occupancy at the landscape-scale based on large-scale sampling blocks, we also investigated use, selection, and habitat characteristics within forest and plantations based on finer scale sampling.

The goals of this study were: 1) to investigate factors affecting tiger probability of occupancy or habitat use, 2) to construct a predictive, spatially-explicit species occurrence model for the forest and plantation landscape in central Sumatra; and 3) to describe habitat characteristics and evaluate the use and selection by tigers between and within different landcover types. We hypothesized that tiger occupancy or habitat use would increase as the proportion of the forested area within the grid cell increases, as the rate of deforestation declines, as altitude declines, as distance to forest centroid and distance to protected area centroid decline, as distance to public roads increases, and as precipitation increases. We also predicted that detection probability would be higher in forests compared to plantation areas.

### Study Area

**Figure 1:**
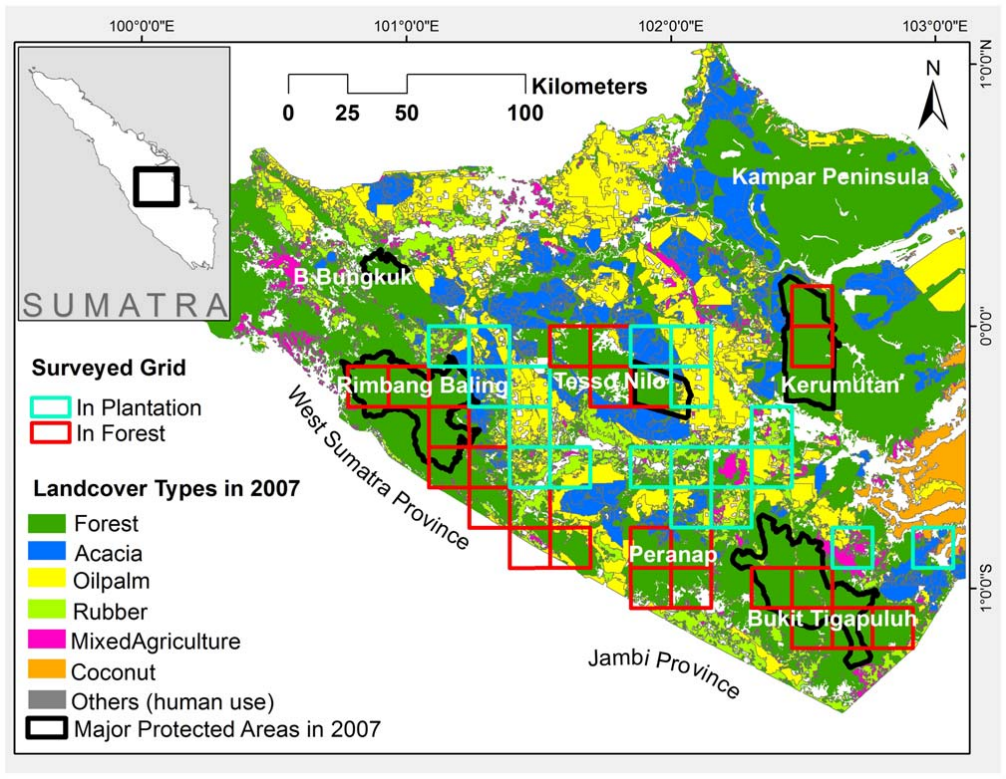
Map of the study area in central Sumatra.

This study was conducted in central Sumatra, covering the southern part of the Riau Province and small portions of Jambi and West Sumatra provinces (Figure 1). The initial survey in this mega-landscape found that Sumatran tigers were distributed in low density, in major protected areas including Tesso Nilo National Park, Bukit Tigapuluh National Park, Rimbang Baling Wildlife Reserve, and Kerumutan Wildlife Reserve and in forests outside of those protected areas [28], [29]. Prior to this survey, tiger presence in plantations such as acacia, oilpalm, and rubber were limited to some anecdotal reports but were never systematically documented.

The land cover in the study area is a mosaic of protected areas, towns and other human settlements, plantations (mainly acacia and oilpalm), mining, and secondary forests [30], [31]. For detailed description of the study area refer to Sunarto (2011) [32]; while detailed study of forest conversion in the area is presented by Uryu et al. [31]. While a relatively large portion of hilly, higher elevation forests are protected, it is not the case with lower elevation areas that include peat-swamp and mineral-soil forests. Examples of unprotected forests include those in Kampar Peninsula, the eastern part of the Kerumutan landscape, the north-western part of the Bukit Tigapuluh landscape, and some areas just outside of Rimbang Baling Wildlife Reserve.

## Results

### Summary of effort

We systematically surveyed 1857 km of transects in 47 17×17 km grid cells covering six different landcover types (Table 1). Each grid cell was surveyed for 40, 1-km transects except in two cells with 29 and 32 km of transects due to logistical constraints. Tiger sign was detected in all but two landcover types: mixed agriculture and coconut plantation.

**Table 1:** Summary of survey effort and detection of tigers in five landcover types in Riau Province, central Sumatra.

	Forest	Acacia	Oilpalm	Rubber	Mixed Agriculture	Coconut	Combined
17×17 km GRID LEVEL							
Number of 17×17 km grid cells surveyed	26	7	6	5	2	1	47
Grid cells with tigers detections	19	3	2	1	0	0	25
Probability of Site Occupancy (ψ_17×17 km_): Naïve estimate*	0.73	0.43	0.33	0.20	0.00	0.00	0.53
1-KM TRANSECT LEVEL							
Number of 1 km transects surveyed	1029	268	240	200	80	40	1857
Transects with tiger detections	81	10	2	1	0	0	94
Probability of Site Use (ψ_1-km_): Naïve estimate	0.08	0.04	0.01	0.01	0.00	0.00	0.05

*number of sites where the species was detected divided by total number of sites surveyed.

### Occupancy models

The best model of tiger-occupancy (ψ_17×17 km_) included 2 variables: altitude (AltDEM) and distance-to-forest-centroid (Table 2). Based on the β estimates, tiger probability-of-occupancy (ψ_17×17 km_) increased strongly with altitude, and decreased, but not strongly with distance-to-forest-centroids (Table 3). Relative estimates of β for every grid-level landscape covariate were consistent in their direction (+/−) in univariate and best models alike (Table 3).

**Table 2:** Top models depicting tiger probability-of-occupancy (ψ_17×17 km_) at the landscape-scale with 17×17 km grid-level landscape covariates in Riau Province, central Sumatra.

Model	AIC	ΔAIC	*w*_i_	Model Likelihood	K
psi(AltDEM+dtf05cr),thta0,thta1; p(LCFor)	318.2	0.0	0.30	1.00	7
psi(AltDEM+dtf05cr+For07Area), thta0, thta1; p(LCFor)	318.9	0.7	0.21	0.69	8
psi(AltDEM),thta0,thta1; p(LCFor)	319.4	1.2	0.16	0.54	6
psi(AltDEM+dtf05cr+For07Area+ dtpacr), thta0,thta1,p(LCFor)	320.7	2.5	0.09	0.28	9
psi(dtf05cr),thta0,thta1,p(LCFor)	321.8	3.6	0.05	0.16	6
psi,thta0,thta1,p(LCFor)	321.9	3.7	0.05	0.15	5
psi(For07Area),thta0,thta1,p(LCFor)	322.0	3.8	0.04	0.14	6
psi(dtpacr),thta0,thta1,p(LCFor)	322.6	4.4	0.03	0.11	6
psi(Def0607),thta0,thta1,p(LCFor)	323.4	5.2	0.02	0.07	6
psi(Precip),thta0,thta1,p(LCFor)	323.8	5.6	0.02	0.06	6
psi(Dtmprd),thta0,thta1,p(LCFor)	323.9	5.7	0.02	0.06	6
psi(.),p(LCFor)	325.6	7.4	0.01	0.02	3
psi,thta0,thta1,p(.)	338.4	20.2	0.00	0.00	4

Psi = probability of site occupancy/habitat use; p = probability of detection; thta0 = spatial dependence parameter representing the probability that the species is present locally, given the species was not present in the previous spatial replicate; thta1 = spatial dependence parameter representing the probability that a species is present locally, given it was present at the previous spatial replicate. AltDEM = Altitude; dtf05cr = Distance to nearest centroid of forest block greater than 50,000 ha; LCFor = Code for forest (1) or non forest (0); For07Area = Area of forest in the grid based on 2007 data; dtpacr = distance to centroid of protected area; Def0607 = Deforested area from 2006 to 2007 in each grid cell; Precip = Precipitation; Dtmprd = Distance to major public road.

**Table 3:** Estimates of β for the logit link function for landscape covariates extracted using GIS based on best and univariate models for tiger probability-of-occupancy (ψ_17×17 km_).

MODEL	Intercept	AltDEM	Dtf05cr	For07Area	Dtpacr	Def0607	Precip	Dtmprd
*A priori* relationship		−	−	+	−	−	+	+
Best(SE)	−7.63(3.938)	*101.09* *(48.941)* *	−0.31(0.220)	NA	NA	NA	NA	NA
Univariate(SE)	NA	*76.72* (41.249)	−0.23 (0.185)	0.18 (0.119)	−0.27 (0.223)	−0.37 (0.490)	0.09 (0.358)	*−0.11* (0.505)

Note:

*indicates strong or robust impact, that is 95% confidence intervals as defined by 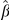
±1.96×SE not overlapping 0; italics indicate opposite from *a priori* prediction. AltDEM = Altitude; dtf05cr =  Distance to nearest centroid of forest block greater than 50,000 ha; For07Area = Area of forest in the grid cell based on 2007 data; Dtpacr = distance to centroid of protected area; Def0607 = Deforested area from 2006 to 2007 in each grid cell; Precip = Precipitation; Dtmprd = Distance to major public road.

#### Spatially explicit occupancy model

**Figure 2:**
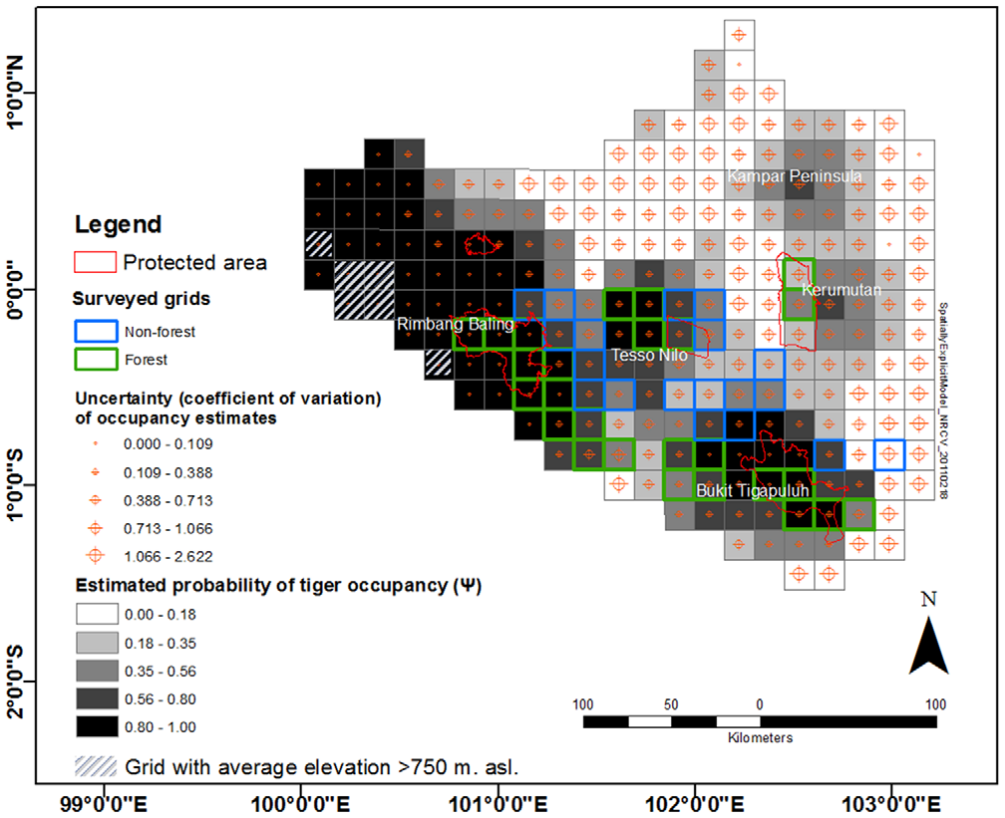
Map of probability of tiger occupancy in the central Sumatra landscape. This map is constructed from the best occupancy model developed based on the landscape-scale survey in 17×17 km grid cells representing forest and other major landcover types.

Using the best model from the model set above, we then developed spatially-explicit predictions of tiger occupancy across the landscape (Figure 2). This prediction shows that sites with higher probability of occupancy were concentrated in the western and southern parts of the study area. The model generally has low confidence (large coefficient of variation) in predicting tiger occupancy in peat swamp areas, which are located in the upper right (NW) of the study area. Models accounting for spatial autocorrelations in detection histories within each site [33], always performed better than original models.

### Habitat use models

#### Across landcover types

The best model included LCCode (corresponding to the distance to and dissimilarity from the forest). This model performed better than those accounting for differences between landcover types as simply categorical (0 or 1). Therefore, we included LCCode as an additional covariate to model p or ψ.

Lumping landcover types together, we found that models including only the LCCode were superior to other models (Table 4a). Estimates of β from the best model for LCCode indicate that probability of use (ψ_1-km_) by tigers strongly decreased as the landcover types increasingly became dissimilar or distant from forest (Table 5). Estimates of β from univariate models under this analysis further indicated that probability of habitat use (ψ_1-km_) increased as altitude, distance-to-freshwater, distance to forest edge, and distance to major public roads increased, and that probability of habitat use (ψ_1-km_) declined as precipitation and distance to centroid of protected areas increased.

**Table 4:** Top models (*w*_i_>0) for tiger probability of habitat use (ψ_1-km_) in central Sumatra across all landcover types in the landscape based on detection history data collected at transect sites (n = 1857, 1-km transects) in six landcover types.

Model	AIC	ΔAIC	*w_i_*	Model Likelihood	K
**a) Developed using landscape covariates**					
psi(LCCode),thta0,thta1,p(LCCode)	1476.97	0.00	0.69	1.00	6
psi(LCCode+altDEM+Precip),thta0,thta1, p(LCCode)	1480.02	3.05	0.15	0.21	8
psi(LCCode+altDEM+Precip+dtwater),thta0, thta1,p(LCCode)	1480.41	3.44	0.12	0.18	9
psi(LCCode+altDEM),thta0,thta1,p(LCCode)	1482.93	5.96	0.03	0.05	7
psi(altDEM),thta0,thta1,p(LCCode)	1499.1	22.13	0.00	0.00	6
**b) Developed using manual covariates**					
psi(understory+LCCode+firerisk+Settlement+Slope+Altitude),thta0,thta1,p(LCCode)	1443.03	0.000	0.52	1.00	11
psi(understory+LCCode+firerisk+Settlement+Slope+Altitude+encroach),thta0,thta1,p(LCCode)	1444.96	1.930	0.20	0.38	12
psi(understory+LCCode+firerisk+Settlement), thta0, thta1,p(LCCode)	1446.16	3.130	0.11	0.21	9
psi(understory+LCCode+firerisk+Settlement+Slope+Altitude+encroach+logging),thta0,thta1,p(LCCode)	1446.62	3.590	0.09	0.17	13
psi(understory+LCCode+firerisk+Settlement+Slope), thta0,thta1,p(LCCode)	1447.00	3.970	0.07	0.14	10
psi(understory+LCCode+firerisk),thta0,thta1,p(LCCode)	1450.78	7.750	0.01	0.02	8

Note: Psi = probability of site occupancy/habitat use; p = probability of detection; thta0 = spatial dependence parameter - probability that the species is present locally, given the species was not present in the previous site; thta1 = spatial dependence parameter -probability that a species is present locally, given it was present at the previous site. LCCode = landcover code; AltDEM = Altitude; Precip = Precipitation; dtwater = distance to freshwater; Dtfedge07 = distance to forest edge; dtf05cr = Distance to nearest centroid of forest block greater than 50,000 ha; dtpacr = distance to centroid of protected area; Dtmprd = Distance to major public road; LCFor = forest(1) or nonforest(0).

**Table 5:** Estimates of β for the logit link function based on best and univariate models for tiger probability of habitat use (ψ_1-km_) in all landcover types in central Sumatra for landscape covariates.

MODEL	Intercept	LCCode	AltDEM	Precip	Dtwater	Dtfedge07	Dtpacr	Dtmprd
*A priori* relationship	NA	**−**	−	+	−	−	−	−
Best(SE)	−3.06 (0.226)	−1.76 (0.304)*	NA	NA	NA	NA	NA	NA
Univariate(SE)	NA	−1.76(0.304)*	*0.17* (0.091)	*−0.19* (0.105)	*0.18* (0.116)	*0.14* (0.104)	−0.13 (0.135)	*0.01* (0.111)

Note:

*indicates strong or robust impact, that is 95% confidence intervals as defined by 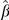
±1.96×SE not overlapping 0; italics indicate opposite from *a priori* prediction. LCCode = landcover code; AltDEM = Altitude; Precip = Precipitation; dtwater = distance to freshwater; Dtfedge07 = distance to forest edge; dtpacr = distance to centroid of protected area; Dtmprd = Distance to major public road.

For the model set based on manual habitat covariates, the best model included understory cover, landcover code (LCCode), fire risk, settlement, slope, and altitude (Table 4b). Based on the parameter estimates for the logit link function, the impacts of understory and altitude were positive and strong, while for landcover code and settlement the impacts were negative and strong (Table 6). Estimates of these covariate parameters, especially in terms of the direction and value relative to the standard error, were also consistent across models.

**Table 6:** Estimates of β for the logit link function based on best and univariate models for tiger probability of habitat use (ψ_1-km_) in all landcover types in central Sumatra for manual covariates.

MODEL	Inter-cept	Under-story	LC-Code	Fire-risk	Settle-ment	Slope	Alti-tude	Encroach	Log-ging	Hun-ting	Sub-canopy	Cano-py	Over-all
*A priori* relationship		**+**	**−**	−	**−**	−	−	−	−	−	+	+	+
Best(SE)	−15.50(3.07)	0.67(0.14)*	−1.28(0.29)*	−0.52(0.32)	−51.89(12.81)*	−0.04(0.15)	*0.32* *(0.13)* *	NA	NA	NA	NA	NA	NA
Univariate(SE)	NA	0.62 (0.12)*	−1.76 (0.30)*	−0.73 (0.31)*	−55.20 (2.12)*	*0.33 (0.11)* *	*4.46 (0.79)* *	−0.29 (0.17)	−0.20 (0.13)	−0.18 (0.16)	0.08 (0.13)	0.07 (0.13)	−0.06 (0.11)

Note:

*indicates strong or robust impact, that is 95% confidence intervals as defined by 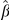
±1.96×SE not overlapping 0; italics indicate opposite from *a priori* prediction. Overall = overall vegetation cover.

**Figure 3:**
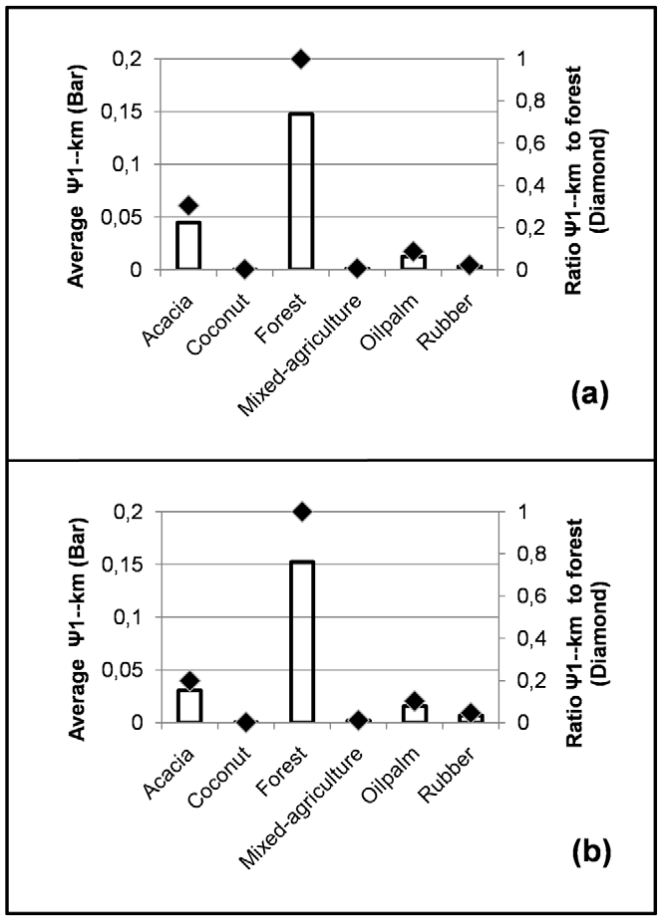
Estimated probability of habitat use (ψ_1-km_) by tigers in six land cover types. These estimates were produced from the best model for each landcover (bars) and ratio of plantation's probability of use (diamonds) relative to forest based on a) landscape covariates and b) manual covariates.

Though slightly different in the value, the ratio of probability-of-use by tigers, relative to forest, consistently decreased with the same rank from acacia, oilpalm, rubber, mixed-agriculture, and coconut both when we model using landscape covariates (Figure 3a) or manual habitat covariates (Figure 3b).

#### Within forest habitat selection

Based on the first set of models developed using landscape covariates, we found that distance-to-freshwater was the single most important variable determining probability of habitat use by tigers within natural forest areas (Table 7a). Tigers strongly selected sites that were farther from water contrary to our *a priori* prediction (Table 8). Furthermore, based on univariate models developed with the rest of the landscape variables, we found that within the forest areas, tigers tended to use areas with higher elevation, lower annual rainfall, farther from forest edge, and closer to forest centroids.

**Table 7:** Top models (*w*_i_>0) for probability of habitat use (ψ_1-km_) by tigers based on detection history data collected at transect sites within forest areas only (n = 1029) in central Sumatra.

Model	AIC	ΔAIC	*w_i_*	Model Likelihood	K.
**a) Developed using landscape covariates**					
psi(dtwater),thta0,thta1,p(.)	1205.18	0.00	0.391	1.000	5
psi(dtwater+Precip),thta0,thta1,p(.)	1205.82	0.64	0.282	0.726	6
psi(Precip),thta0,thta1,p(.)	1208.98	3.80	0.058	0.150	5
psi,thta0,thta1,p(.)	1209.01	3.83	0.058	0.147	4
psi(dtf05cr),thta0,thta1,p(.)	1209.35	4.17	0.049	0.124	5
psi(Dtfedge07),thta0,thta1,p(.)	1209.71	4.53	0.041	0.104	5
psi(altDEM),thta0,thta1,p(.)	1209.82	4.64	0.038	0.098	5
psi(dtwater+dtf05cr),thta0,thta1,p(.)	1210.31	5.13	0.030	0.077	5
psi(Dtmprd),thta0,thta1,p(.)	1210.87	5.69	0.023	0.058	5
psi(dtpacr),thta0,thta1,p(.)	1210.98	5.80	0.022	0.055	5
1 group, Constant P	1212.94	7.76	0.008	0.021	2
**b) Developed using manual covariates**					
psi(understory+encroach+Settlement+Slope), thta0,thta1,p(.)	1172.06	0.00	0.384	1.000	8
psi(understory+encroach+Settlement+Slope+firerisk+Altitude),thta0,thta1,p(.)	1172.62	0.56	0.290	0.756	10
psi(understory+encroach+Settlement+Slope+firerisk),thta0,thta1,p(.)	1174.05	1.99	0.140	0.370	9
psi(understory+encroach+Settlement+Slope+firerisk+Altitude+Hunting),thta0,thta1,p(.)	1174.58	2.52	0.110	0.280	11
psi(understory+encroach+Settlement),thta0, thta1,p(.)	1176.25	4.19	0.050	0.120	7
psi(understory+encroach),thta0,thta1,p(.)	1177.34	5.28	0.030	0.070	6
psi(understory),thta0,thta1,p(.)	1190.35	18.29	0.000	0.000	5

Notes: Psi = probability of site occupancy/habitat use; p = probability of detection; thta0 = spatial dependence parameter representing the probability that the species is present locally, given the species was not present in the previous site; thta1 = spatial dependence parameter representing the probability that a species is present locally, given it was present at the previous site. Dtwater = distance to freshwater; Precip = precipitation; dtf05cr = Distance to nearest centroid of forest block greater than 50,000 ha; dtfedge07 = distance to forest edge; altDEM = altitude; Dtmprd = distance to major public road; dtpacr = distance to centroid of protected areas.

**Table 8:** Estimates of β for the logit link function based on best and univariate models for tiger probability of habitat use (ψ_1-km_) within forest areas in central Sumatra for landscape covariates.

MODEL	Intercept	AltDEM	Precip	Dtwater	Dtfedge07	Dtpacr	Dtmprd	dtf05cr
*A priori* relationship	NA	−	+	−	+	−	−	−
Best(SE)	−1.808 (0.195)	NA	NA	*0.289 (0.120)* *	NA	NA	NA	NA
Univariate(SE)	NA	*0.130*(0.117)	*−0.178*(0.125)	*0.289* *(0.120)* *	0.135(0.117)	*0.021*(0.124)	−0.048(0.130)	−0.1670(0.132)

Note:

*indicates strong or robust impact, that is 95% confidence intervals as defined by 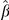
±1.96×SE not overlapping 0; italic indicates opposite from *a priori* prediction. AltDEM = altitude; Precip = precipitation; Dtwater = distance to freshwater; dtfedge07 = distance to forest edge; dtpacr = distance to centroid of protected areas; Dtmprd = distance to major public road;dtf05cr = Distance to nearest centroid of forest block greater than 50,000 ha.

Based on models developed using manual covariates, we found four variables (understory cover, encroachment, settlement, and slope) to be the most important factors determining tiger probability of habitat use within forest areas (Table 7b). All of those variables had strong effects on tiger probability of habitat use (Table 9). Tigers strongly preferred forest with denser understory cover and steeper slope, and they strongly avoided forest areas with higher human influence in the forms of encroachment and settlement. We found that accounting for slope in modeling detection probability produced models that performed better than the best *a priori* model (delta AIC = 5.23), which accounted for slope in the probability-of-occupancy instead of detection. Beta estimates (β[SE]) from this new model for slope as a detection covariate was 12.25 (4.59) meaning that the probability of detecting tigers strongly increases with slope.

**Table 9:** Estimates of β for the logit link function based on best and univariate models for tiger probability of habitat use (ψ_1-km_) within forest areas in central Sumatra for manual covariates.

MODEL	Inter-cept	Overall	Subca-nopy	Under-story	Logging	Encroach	Fire-risk	Settle-ment	Hunting	Altitude	Slope
*A priori* relationship	NA	**+**	**+**	**+**	**−**	**−**	**−**	**−**	**−**	**−**	**−**
Best(SE)	−18.047 (1.954)	NA	NA	0.652(0.140)*	NA	−0.769(0.350)*	NA	−88.89 (10.850)*	NA	NA	*0.33 (0.15)* *
Univariate(SE)	NA	*−0.173* *(0.125)*	*−0.002* *(0.127)*	0.582(0.135)*	−0.159(0.1459)	−0.742(0.330)*	−0.623(0.352)	−160.134(7.278)*	−0.198(0.132)	0.205(0.118)	*0.359* *(0.127)* *

Note:

*indicates strong or robust impact, that is 95% confidence intervals as defined by 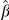
±1.96×SE not overlapping 0; italic indicates opposite from *a priori* prediction.

#### Within acacia plantation habitat selection

We found distance-to-freshwater and distance-to-major-public-road as the most important variables determining tiger probability of habitat use within acacia plantations (Table 10a). In contrast to forest areas however, within acacia plantations tigers tended to use areas closer to water (Table 11).

**Table 10:** Top models (*w*_i_>0) for probability of habitat use (ψ_1-km_) by tigers based on detection history data collected at transect sites within acacia plantations (n = 268, at 1-km transect scale) in central Sumatra.

Model	AIC	ΔAIC	*w_i_*	Model Likelihood	K
**a) Developed using landscape covariates**					
psi(dtwater+Dtmprd),thta0,thta1,p(.)	198.15	0.00	0.395	1.000	6
psi(dtwater+Dtmprd+Dtfedge07),thta0,thta1,p(.)	198.68	0.53	0.303	0.767	7
psi(dtwater),thta0,thta1,p(.)	199.15	1.00	0.240	0.607	5
psi,thta0,thta1,p(.)	205.07	6.92	0.012	0.031	4
psi(Dtmprd),thta0,thta1,p(.)	205.24	7.09	0.011	0.029	5
**b) Developed using manual covariates**					
psi(Slope+subcanopy+encroach+logging), thta0,thta1,p(.)	182.66	0.00	0.543	1.000	8
psi(Slope+subcanopy+encroach+logging+firerisk),thta0,thta1,p(.)	183.49	0.83	0.359	0.660	9
psi(Slope+subcanopy+encroach),thta0,thta1,p(.)	186.29	3.63	0.090	0.163	7
**c) Developed using manual plantation-specific covariates**					
psi(Age+HumanActivities+LeafLitter), thta0,thta1,p(.)	174.91	0.00	0.358	1.000	7
psi(Age+HumanActivities+LeafLitter+TreeHeight),thta0,thta1,p(.)	175.42	0.51	0.278	0.775	8
psi(Age+HumanActivities),thta0,thta1,p(.)	176	1.09	0.208	0.580	6
psi(Age+HumanActivities+LeafLitter+TreeHeight+OtherPlants),thta0,thta1,p(.)	176.73	1.82	0.144	0.403	9
psi(Age+LeafLitter),thta0,thta1,p(.)	182.77	7.86	0.007	0.020	6

Notes: Psi = probability of site occupancy/habitat use; p = probability of detection; thta0 = spatial dependence parameter representing the probability that the species is present locally, given the species was not present in the previous site; thta1 = spatial dependence parameter representing the probability that a species is present locally, given it was present at the previous site. Dtwater = distance to freshwater; Dtmprd = distance to major public road; dtfedge07 = distance to forest edge; dtpacr = distance to centroid of protected areas; dtf05cr = Distance to nearest centroid of forest block greater than 50,000 ha; Precip = precipitation.

**Table 11:** Estimates of β for the logit link function based on best and univariate models for tiger probability of habitat use (ψ_1-km_) within acacia plantations in central Sumatra for landscape covariates.

MODEL	Intercept	AltDEM	Precip	Dtwater	Dtfedge07	Dtpacr	Dtmprd	dtf05cr
*A priori* relationship	NA	−	+	−	−	−	+	−
Best(SE)	−3.242 (0.885)	NA	NA	−1.160 (0.442)*	NA	NA	*−0.773 (0.440)*	NA
								
Univariate(SE)	NA	NA	0.347(0.378)	−2.715(2.145)	−0.522(0.456)	−0.455(0.405)	*−0.552*(0.462)	−0.410(0.365)

Note:

*indicates strong or robust impact, that is 95% confidence intervals as defined by 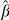
±1.96×SE not overlapping 0; italics indicate opposite from *a priori* prediction. AltDEM = altitude; Precip = precipitation;Dtwater = distance to freshwater body; dtfedge07 = distance to forest edge; dtpacr = distance to centroid of protected areas; Dtmprd = distance to major public road; dtf05cr = Distance to nearest centroid of forest block greater than 50,000 ha.

Using manual covariates, we found four variables (slope, sub-canopy cover, encroachment, and logging) to be the most important factors determining habitat use by tigers (Table 10b). Of these four variables only logging had a strong impact (Table 12) with tigers avoiding areas with higher logging activity, and avoiding steeper areas. In acacia plantations, tigers preferred areas with thicker sub-canopy cover and with higher level/risk of encroachment.

**Table 12:** Estimates of β for the logit link function based on best and univariate models for tiger probability of habitat use (ψ_1-km_) within acacia plantations in central Sumatra for manual covariates.

Model	Intercept	Overall	Canopy	Sub-canopy	Under-story	Logging	En-croach	Fire-risk	Settle-ment	Hun-ting	Altitude	Slope
*A priori* relationship	**+**	**+**	**+**	**+**	**−**	**−**	**−**	**−**	**−**	**−**	**−**	
Best	−59.47	NA	NA	1.53	NA	−128.65	*2.32*	NA	NA	NA	NA	−2.36
(SE)	(1.986)			(1.397)		(0.853)*	(1.43)					(1.73)
												
Univariate	NA	0.46	0.46	7.33	0.15	−153.81	*3.25*	−1.14	−32.94	*0.43*	*0.16*	−4.97
(SE)		(0.559)	(0.559)	(12.59)	(0.342)	(1.255)*	*(5.062)*	(0.89)	(0.415)	*(0.391)*	*(0.351)*	(4.994)

Note:

*indicates strong or robust impact, that is 95% confidence intervals as defined by 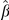
±1.96×SE not overlapping 0; italics indicate opposite from *a priori* prediction.

Based on covariates collected in plantation areas only, three variables (plant age, human activity, and leaf litter) were found to be the most important in determining tiger habitat use in acacia plantations (Table 10c). Tigers preferred areas with older plants and more leaf litter; and avoided areas with high human activity. Estimates of β from univariate models show that tigers strongly preferred areas with taller trees, and strongly avoided areas with higher intensity of plantation management activity (Table 13).

**Table 13:** Estimates of β for the logit link function based on best and univariate models for tiger probability of habitat use (ψ_1-km_) within acacia plantations in central Sumatra for plantation-specific manual covariates.

MODEL	Intercept	Age	TreeHeight	Hus-bandry	OtherPlants	LeafLitter	HumanActivities	PlantIntervals	Rota-tion
*A priori* relationship	NA	**+**	**+**	**−**	**+**	**+**	**−**	**+**	**−**
Best	−8.08	3.26				2.02	−3.65		
(SE)	(2.98)	(1.83)	NA	NA	NA	(1.35)	(2.51)	NA	NA
Univariate(SE)	NA	7.25(4.864)	2.74(1.284)*	−0.97(0.453)*	3.36(1.725)	8.01(5.767)	NA	*−0.15*(0.314)	*0.001*(0.337)

Note:

*indicates strong or robust impact, that is 95% confidence intervals as defined by 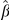
±1.96×SE not overlapping 0; italics indicate opposite from *a priori* prediction.

We descriptively summarized the few records of tiger detections from oilpalm and rubber plantations as they provide some rare evidence on the use of such areas by tigers. In oilpalm plantations, tiger sign was detected only in two locations that were measured respectively ∼13 and ∼7.5 km from the edge of the nearest large (>50,000 ha) forest block. The only record of tiger sign in the rubber plantations was documented in a site that was ∼16 km away from the edge of the nearest large forest blocks.

## Discussion

Considering the dynamic nature of tiger landscapes in Sumatra and elsewhere, it is crucial to understand spatial patterns of tiger occupancy and habitat use across the spectrum of habitat types. This paper provides information important for current and future management of tigers and other wide-ranging carnivores living in landscapes that are increasingly dominated by humans, particularly in South East Asia.

This paper is unique in that it describes how to use an occupancy analysis approach on two different scales simultaneously - at the macro-habitat level (similar to the Wibisono et al. [34] approach) and at the micro-habitat scale for habitat use within a tiger's home range. While Wibisono et al. provide information on occupancy with partial contribution of data from this study, the results cannot be compared directly with this study due to the differences in some critical aspects (such as landcover types surveyed, geographic coverage, and covariates used in the models that are different and specific to this study).

### Tiger occupancy and habitat use in central Sumatra

#### Scale independent factors

Understory cover was consistently found to have positive impacts on tiger probability of occupancy and habitat use across the landscape and within different types of landcover. This suggests that availability of adequate vegetation cover at the ground level served as an environmental condition fundamentally needed by tigers regardless of the location. Without adequate understory cover, tigers, as an ambush hunter [1], would find it hard to capture their prey, even if prey animals are abundant. Furthermore, without adequate understory cover, tigers are even more vulnerable to humans who generally perceive them as dangerous and readily persecute them. Although this likely applies to all tigers, it is particularly relevant to Sumatran tigers. Perhaps human persecution of tigers [35], [36] has become an important selection factor contributing to the overall secretive behavior of tigers in the region, causing this obligatory requirement for ample understory cover.

Variables that represent distance or dissimilarity from forest such as landcover rank and distance to centroid of forest block greater than 50,000 ha, also consistently negatively and strongly impacted tiger occupancy and/or habitat use. These results indicate that although tigers were capable of using some plantation areas, especially acacia, forest remained their core habitat without which they are unlikely to survive in Sumatra.

In other parts of Sumatra such as Aceh [37], tigers and several other animal species were found to be very sensitive to human activities. We found that human-disturbance-related variables negatively affected tiger occupancy and habitat use. However, the effects of these variables were not always strong. Those variables with strong impacts include a) ‘settlement’ in the best and univariate habitat use models, both within forest areas and across six landcover types; b) ‘encroachment’ in the best and univariate models for tiger habitat use within forest areas; c) ‘logging’ in best and univariate models for habitat use within acacia plantations; and d) ‘husbandry’ (the intensity of maintenance for plantation to be productive) in univariate model for habitat use within acacia plantations.

Human disturbance can take different forms in different landcover types. In forest areas, sites with a large encroachment score had higher levels of human activity, which was not always the case in plantations. In acacia plantations, areas with higher encroachment scores were typically those that had lower levels of plantation care management activities and could actually have lower levels of human activity. This typically happened in areas considered by plantation managers to be less productive such as areas with unresolved land status. Highly encroached acacia plantations, therefore, did not necessarily have higher levels of human activity.

#### Scale dependent factors

The impact of altitude on tiger occupancy or habitat use depended on the scale and context of analysis. We found that, overall, probability of tiger occupancy increased with altitude, but, within forest areas, the impacts were not as strong for both landscape variables and manual variables. Meanwhile, in another island-wide analysis [34], tiger occupancy (within forest) is higher at lower altitude. In acacia plantations, the model failed to converge when we used altitude as a landscape variable, and had a small estimated impact when we used altitude data from manual variables.

We suspect that altitude, which is strongly correlated with slope, was negatively correlated with overall human activity. In our study area, human activities affecting tiger habitats (such as conversion of forests into plantations), generally occur in flat lowland areas, followed by either swampy/peatland areas or hilly areas. The later are generally considered less suitable for plantations especially oilpalm. Most of the remaining forests, particularly those growing on mineral soils, are at higher altitudes. Because of the high demand for flat land at low elevation, forests in such areas were degraded at a much faster rate and therefore predicted to go extinct sooner than forest at higher altitudes [38].

The importance of altitude/slope on tiger occupancy is also driven by the fact that peat swamps dominated the low-lying forest types in the landscape. Such forest types are lower in quality compared to mineral soils forests and have low levels of primary productivity [39]. They do not support high ungulate community biomass likely because ungulates with pointed feet face difficulties travelling in such terrains with soft ground and porous texture. Previous work [32] documented an extremely low abundance of potential prey in peat land areas. Finally, post-hoc models including slope for tiger detection probability, instead of occupancy, were superior based on AIC rankings. This suggested that within forest areas tigers are more easily detected in steep-terrain. Therefore it is important to determine if tigers preferred such areas, or rather that detection was easier due to funneling animals along the strip of a narrow of ridge or valley.

Distance-to-freshwater had strong yet inconsistent impacts on tiger probability-of-use in different landcover types. For forest area, it is likely that water acts as a proxy for human activities as people tend to concentrate around water bodies. For other landcover types such as acacia where areas surrounding water bodies or riparian areas are (supposed to be) protected, the relationship between water bodies and human activities might be the opposite or not as strong. Water availability is not likely a critical issue for tigers in the landscape. This region already has relatively high annual rainfall (more than 2210 mm/year from 2004 to 2006). Additionally most sites within the study area had a dense network of streams in the upper lands, or wider rivers and other water bodies such as lakes or swamps in the low lands.

#### Non-influential factors

Variables that were never identified as important at any scale include “canopy”, “sub-canopy”, and “overall” vegetation cover in all landcover types, and “rotation” and “plant interval” in plantation areas. The fact that “rotation” and “plant interval” did not impact tiger use in this study was most likely due to low variation in these variables. Except for understory, no vegetation-related characteristics were important determinants of tiger use. This result suggests that, with other factors (particularly human disturbance) being equal, tigers not only used but seemed to prefer forests that were selectively logged or slightly disturbed, as they tended to have thicker understory cover compared to mature primary forest. Therefore, restoration of previously disturbed or logged forests should not focus on achieving ‘climax’ primary forest condition. Instead, reducing the level of human disturbance and maintaining adequate understory cover would likely be more beneficial for tigers.

### Landscape-scale assessment: occupancy models and spatially-explicit predictions

At the landscape-scale, closely-competing models included proportion of forest within grid cells and distance-to-centroid-of-protected-area, in addition to altitude. Distance-to-road, which was identified as the most important factor representing human disturbance in a previous study in the neighboring landscape of Kerinci-Seblat [23], was not an important factor in this study. However, landscape characteristics such as variation in forest type, extent and relative position of landscape features (especially public roads relative to forest blocks) appear to be very different between the Kerinci-Seblat and our study area.

Importantly, based on the spatially-explicit model, many areas with high probability of tiger occupancy were located outside of existing protected areas. Areas with high estimated probability of occupancy and with high precision (low coefficient of variation) were concentrated in the southwestern part of the landscape. Our landscape model predicted higher elevation areas to have a higher probability of tiger occupancy, even after excluding those areas with values well beyond the range of surveyed altitude values. Our model predicted relatively large areas with very high probability of tiger occupancy, particularly to the northwest of Rimbang Baling Reserve. In contrast, although with lower precision, current protected areas in peat land (i.e., Kerumutan Wildlife Reserve/KWR) had little area with a high probability of tiger occupancy. Interestingly, areas with the highest probability of occupancy in peat land are currently not protected. These include areas east of KWR, on the Kampar Peninsula, and on the western part of Bukit Tigapuluh. In fact, large portions of these areas were proposed for, or are already in the process of, conversion by either pulp-and-paper- or palm-oil-producing companies [40], [41].

More intensive surveys are required to obtain better precision in occupancy estimates in peat land areas. Low occupancy with large coefficient of variation could result from both ecological factors (low abundance) and survey difficulties (logistical challenges and low detectability). Further surveys and alternative methods that can overcome the study challenges in such a poorly studied habitat type should be explored. For example, it might be possible to improve sign detection by using baited track stations [42] or trained scat-detector dogs [43] to assist in sign detection of target species.

The spatially-explicit model we developed could serve as the basic framework for developing a tiger conservation vision at the mega-landscape-scale. To maximize the likelihood of success in tiger conservation, priorities should be directed toward securing those areas with highest probability of occupancy through protection and better management. Critical areas, for example those crucial for connectivity between two closely-located habitat blocks, could also be identified and managed to allow tiger dispersal [32].

### Between and within landcover type: habitat characteristics, use and selection

In estimating tiger probability of use, different models consistently ranked plantations in the following order from best to worst: acacia, oilpalm, rubber, mixed-agriculture, and coconut. Such a ranking system is useful for tiger conservation, but should be considered within the context of the landscape studied rather than generalized to other study areas. For example, while vegetation characteristics did play a role in determining occupancy and habitat use by tigers, so did other characteristics such as plantation age, historical impacts, managerial aspects of plantations, and extent and configuration of a particular type of plantation in the landscape in relation to proximity to forest blocks.

With context and scale recognized, rank can be used to prioritize the types of plantations in the landscape that should be managed to improve tiger conservation. For example, timber/pulp-and-paper plantations such as acacia could be improved as tiger habitat by regulating/reducing the level of human activities and improving the vegetation that benefit tiger prey as well as cover for tigers to hunt. Each type of plantation should be able to facilitate the movement of tigers between patches of forest, and prey animals were available in most areas, including plantations where signs of wild boar were commonly found.

Certain individual animals - possibly sub-adult transients - did venture through plantations relatively far (up to ∼16 km) from core forest habitat areas. Likely factors that motivated dispersal include the ‘push’ from the territorial-holding adults and ‘pull’ from the availability of habitable spaces, prey, and possibly mates in other places [44], [45]. Such movements likely were facilitated by the existence of riparian areas in the study area that served as corridors, the availability of small patches of forests that served as sort of ‘stepping stones’, and the mosaic of plantations with adequate understory cover that provide habitat connectivity.

### Implications

This study highlights the importance of scale and context in the assessment of tiger habitat use. For example, altitude can have different impacts depending on the analysis scale and the importance of distance to freshwater depended on the landcover type. Tiger management therefore, should correspondingly consider scale and context in restoration efforts. Once a broad-scale vision, for example tiger conservation at the mega-landscape, has been clearly defined, management goals can then be identified for specific areas at finer-scales. Considering the dynamic nature of the landscape, it is important to continuously evaluate the landscape conditions, including land conversion and plantation age and iteratively adjust conservation strategies and goals.

Although the overall value for tigers of any plantation was much lower than forests, management practices can be adjusted so tigers can still use them without necessarily causing negative economic impacts. Management plans should include existing plantations, particularly those that are forestry-based such as acacia which possessed high potential to be reclaimed for additional tiger habitat or as corridors, stepping stones, or mosaics of connectivity facilitating animal movement [46]. In addition, plantation concessions that border protected areas can potentially serve as buffers to reduce human disturbance and provide additional forest protection.

While prey animals appear to be available across plantation areas, the most basic requirements generally lacking from plantations were adequate understory cover and low levels of human activity. If these two factors can be improved, especially radiating out from the main forest habitat, tigers likely will use these areas. Concurrently human-tiger conflict should be minimized through awareness, training, and education programs designed to build understanding and appreciation for wildlife among local people. If such an initiative is replicated across the landscape, tiger recovery is possible.

## Methods

This study is part of a collaborative project among World Wildlife Fund (WWF), Virginia Tech, and the Indonesian Ministry of Forestry to conserve the Sumatran tiger. Field surveys were conducted under the support from the Director of Biodiversity Conservation, Indonesian Ministry of Forestry (Letter # S.784/IV/KKH-I/2007).

We used occupancy modeling techniques [18], [19], [20], [21] to investigate the influence of biotic and abiotic factors on tigers' large-scale selection of homerange (occupancy at 17×17 km grid cells) and fine-scale selection of habitat (habitat use in 1-km transects) in forest and plantation areas. Partial data from this study have been contributed to the island-wide tiger assessment [34].

### Sampling design

We superimposed the entire study area with a 17×17 km grid and selected 47 (∼15% of the total area) grid cells in which to conduct detection, non-detection surveys. The grid cell size was selected because it approximated the home-range size of tigers in low density areas [4], [47]. We used the landcover classification available from WWF-Indonesia [48] to stratify the sampling by landcover type based on the proportion of availability across the landscape.

We conducted detection, non-detection sampling at two spatial scales simultaneously. At the landscape-level, each site was represented by the 17×17 km grid cell, while sampling occasions within each site were represented by ∼40, 1-km transects. At a finer scale, we considered each 1-km transect as the site, and sampling occasions were represented by ten 100-m segments. This approach resembles ‘robust design’ in capture-mark-recapture studies as described by Pollock et al. [49]. As such, it is possible to estimate not only the tiger's probability of occupancy at the 17×17 km grid level, but also probability of habitat use at a finer scale based on observations conducted at 1-km transect level [33].

To minimize observer bias and spatial autocorrelation in selecting the survey area, we applied two levels of randomization. First, we randomly selected a 2×2 km sub-cell within each 17×17 km grid cell to conduct transects for each team. When more than one team covered the same 17×17 km grid cell, we selected an independent random 2×2 km sub-cell for each team. Second, we randomized the transect start-point by walking 200 m following a random azimuth from the end point of the previous transect or drop point from the last vehicle access. Field testing of this approach indicated that, in most situations, observers lacked the ability to predict what the conditions were like beyond 200-m from the previous end point. We believe this technique minimized observer bias in selecting areas to survey and minimized autocorrelation between consecutive transects. Further attempts to mitigate the impacts of spatial autocorrelation were done at the analysis stage detailed below.

Along transects the team surveyed areas deemed to have the highest likelihood of finding tiger sign (tracks, scat, scrapes). The team intensively searched for tiger and prey signs on forest trails, sand beds, river banks, ridgelines, and other areas where tiger sign were likely to be found. We did this, instead of following a straight line as typically done in Distance Sampling [50], because of the infeasibility and ineffectiveness of tiger sign sampling following straight lines, proven through preliminary method-testing.

### Animal sign and environmental/habitat conditions

Each 1 km transect was divided into ten, 100-m segments where sign surveys and environmental variables were noted and measured in every segment. At the level of 17×17 km grid cell we used Geographic Information System (GIS) software ArcGIS version 9.3.1, to extract grid-level landscape variables described below. For every 1-km transect, the team recorded the weather and GPS coordinates for the start and end of each transect. At every 100-meter segment we measured altitude using the barometric altimeter available in Garmin® GPS units, and we tallied the scores for overall vegetation cover, canopy cover, sub-canopy cover, understory cover, and slope (Appendix S1). We also observed, assessed, and scored the impact and/or risk of logging, encroachment, fire, settlement, and hunting on a 1–5 scale.

Due to the uniqueness of plantation characteristics, we assessed additional variables for plantations collected at every 100-m segment that included estimates of tree age, tree diameter and height, intensity of plant husbandry, the presence or cover of plants other than the main commodity species, leaf-litter cover, level of human activity, interval/distance between individual plants, and planting rotation (Appendix S2). We summarized the values from observations of every habitat variable conducted at 100-m segments and treated them as site covariates associated with each 1-km transect.

A guideline developed based on field-testing was used to assist observers in assigning habitat scores. When ambiguities in assigning scores were found between observers, we averaged the values from all observers. We believe the variable scores we documented effectively depicted habitat conditions while being relatively practical to collect and can be used for rapid assessment with relatively low levels of training.

At each detection we recorded species, sign type (e.g., direct sighting, track, scat/dung), distances from the start point of transect, and perpendicular distance from the center of the transect line. Detections of multiple signs from the same species in the same segment were noted as only a single detection. For this paper, we focus on tigers and reduced all such information into whether or not the animal was detected in a given segment (for analysis at finer-scale) or transect (for analysis at landscape-scale).

Environmental variables at the landscape level were extracted from GIS layers available from World Wildlife Fund (WWF) Indonesia GIS Unit. These include landcover [48]; roads (updated from original data from Indonesian national survey and mapping coordinating agency - BAKOSURTANAL); and boundaries of conservation areas, boundaries of forestry concessions, and boundaries of agricultural concessions. We also obtained several GIS layers available in the public domain such as Bioclim interpolated precipitation version 1.4 [51], freshwater (rivers, canals, and lakes data) from Digital Chart of the World downloaded through http://www.diva-gis.org/gData, and Digital Elevation Model/DEM from Shuttle Radar Topographic Mission/SRTM version 4 available from International Center for Tropical Agriculture/CIAT [52]. A complete list of landscape variables derived from GIS, the original source, and treatments to the data are presented in Appendix S3.

### Analyses

We used Program PRESENCE version 2.4 [53] to estimate the probability of occupancy (ψ_17×17 km_) across the 17×17 grid cells and to assess habitat use by tigers within each landcover type where adequate tiger detection data were collected, based on the probability of use (ψ_1-km_) at the finer spatial scale. PRESENCE uses the models developed by MacKenzie et al. [18], [19] and others such as Hines et al. [33] for the spatial autocorrelation model, to estimate the probability-of-occupancy or probability-of-use from detection-non-detection data collected in a series of patches, sites, and/or grid cells.

We modeled the effects of different covariates (i.e., landscape, habitat, and environmental variables) on tigers' probability of occupancy (ψ_17×17 km_) or habitat use (ψ_1-km_). At the landscape level, we treated each 17×17 km cell as the ‘site’, while each 1-km transect represented a sampling occasion or replicate. As the 17×17 km grid size was considered close to the animal's home-range size, the landscape-scale analyses were therefore expected to reveal the ‘true occupancy’ for tigers. To investigate the tiger's habitat-use within certain landcover types, we considered each1-km transect as the ‘site’ while the 100-meter segments represented ‘replicates.’

We explored the correlations between environmental/habitat variables and eliminated highly correlated variables to reduce the number of covariates used in occupancy analyses. We considered variables highly correlated when correlation coefficients were higher than 0.6 [54], [55]. For highly correlated variables, we selected the one considered most representative based on its ecological relevance, availability across wider area, ease of collection, or a combination of these.

#### Developing a spatially-explicit landscape-scale tiger occupancy model

Preliminary analysis revealed that collapsing the detection/non-detection history data from 40 to 10 occasions reduced the number of zeros in the data and stabilized the numerical algorithms used in Program Presence. Therefore, each occasion represents 4 consecutive 1-km transects within each 17×17 km site.

Due to the relatively low number of samples at the landscape-level (i.e., 47, 17×17 km sites), it was not feasible to include all available GIS covariates in the occupancy model [54], [56]. Hence, considering the correlations between variables (Appendices S4, S5, S6, S7) and using *a priori* knowledge related to tiger ecology, we ensured that the number of covariates used in the models was no more than 20% of the number of sites.

Following the above procedure, we retained 7 GIS-based landscape-scale continuous variables (hereafter ‘grid-level landscape variables’) used to model the probability of occupancy (ψ_17×17 km_). The variables were: 1) forest area within each grid cell based on the condition in 2007 (“For07Area”), 2) rate of deforestation from 2006 to 2007 for each 17×17 km grid cell (“Def0607”), 3) altitude based on Digital Elevation Model/DEM (“AltDEM”), 4) distance to forest centroids (defined as the centroid of contiguous forest area equal to or greater than 50,000 ha based on conditions in 2005; “DtF05Cr”), 5) distance to the centroid of protected area (“Dtpacr”), 6) distance to major public road (“Dtmprd”), and 7) interpolated precipitation averaged for each grid cell (“Precip”). In addition, we also used the incremental scoring (1 to 6) of landcover type according to its dissimilarity from forest (“LCCode”) or forest/non forest category (“LCFor”) to model the detection probability of the animal. Records for the seven continuous variables are available for all the grid cells across the landscape; while the categorical landcover type or forest/non-forest variable is available for only the surveyed grid cells. Values for each variable were normalized and/or scaled by computing z-values (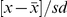) as a means of covariate transformation, while scaling was done by dividing the covariate value by a constant (Jim Hines/USGS, pers. comm.).

In building each model set for occupancy (ψ_17×17 km_), first we entered each variable in a univariate model as a function of the above listed seven variables. For each case, we modeled the detection probability (*p*) as either constant (.) or influenced by two-category landcover type (LCFor) coded as forest (1) or non-forest (0). Based on the performance of the univariate models we constructed multivariate models using combinations of covariates and included at least one of those covariates that performed relatively well in univariate models, similar to the approach suggested by Thomas et al. [57]. Models were ranked and evaluated based on Akaike Information Criteria (AIC) [58]. We considered models to be competing if they were within 2 delta-AICs of the top model and models with some support if they were between 2–4 delta AICs. To evaluate and mitigate the impact of spatial autocorrelation in the detection history data on the parameter estimates, we also ran custom models incorporating spatial autocorrelation [33].

We selected the best model based on AIC and used the estimates of probability of occupancy for each grid cell to construct a spatially-explicit tiger distribution model across the landscape, including the un-surveyed areas. We evaluated the uncertainty in the estimates based on the coefficient of variation (standard errors divided by the occupancy estimate) for each cell. In the resulting map depicting probability of tiger occupancy across the landscape, we highlighted un-surveyed cells that have covariate values far beyond the range of the surveyed cells. For example, although actual surveys were conducted in elevations ranging from 0 to 1,250 m (measured directly in the transect), the highest average elevation of a surveyed cell was below 500 m above sea level (based on DEM calculated in GIS); hence we had little confidence in the prediction of tiger occupancy for grid cells that had average elevations far beyond that range and we highlighted cells where average elevation was higher than 750 m.

#### Assessing tiger use- and selection-of-habitats within forest and plantation areas

Estimates of the occupancy or probability of use at the patch, or within-habitat, scale are similar to the resource selection function (RSF) or, depending on the sampling design, resource selection probability function (RSPF) [59], [60]. But, traditional methods of estimating the RSPF are based on presence-absence data assuming that a non-detection is an absence. Therefore, occupancy techniques can produce more accurate estimates of the probability of habitat use by incorporating detectability.

Our fine scale sample units were represented by 100 m segments of the 1-km transects, augmenting the number of samples by an order of magnitude, allowing us to investigate the tiger's use- or selection-of-habitat within selected landcover types containing adequate records of tiger detections. We developed habitat use (ψ_1-km_) models using different sets of covariates including a) variables extracted in GIS from 500-m radii of the start- and end-point of each 1-km transect (values from both circles were averaged), hereafter ‘landscape covariate’ b) variables scored directly in the field and tallied in each transect in all landcover types, hereafter ‘manual covariates’ and c) specific variables observed and tallied only in plantation areas, hereafter ‘manual plantation-specific covariates.’ We also combined sets of covariates to model tiger habitat use (ψ_1-km_) either for 1) all landcover types, 2) forest only, or 3) specific plantations where adequate tiger detections were obtained (i.e., acacia).

Within each landcover-type, habitat use models were developed for natural forest and acacia plantations only. We could not develop models for other landcover types due to the small number of tiger detections. For those, we focused on qualitative rather than quantitative analysis.

We developed habitat use models by incorporating the effect of different covariates with similar procedures used to develop the landscape level occupancy models. We extracted the estimates of the probability-of-habitat-use (ψ_1-km_) from the best models to calculate the likelihood ratios of habitat use between landcover types. Meanwhile, we also used the untransformed estimates of coefficients for covariates (β) to evaluate the effect of different variables on the probability of habitat use (ψ_1-km_) by tigers. We considered a covariate to have a strong or robust impact on ψ if its respective estimate of β has 95% confidence limits (calculated as 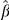
±1.96×SE) that did not include zero [61].

## Supporting Information

Appendix S1 List of habitat and environmental variables (manual covariates) collected in every 100-m segment along 1-km transects in forest and plantation landscapes of central Sumatra.
(DOC)

Appendix S2 Additional environmental variables (manual plantation-specific covariates) collected in every 100-m segment along transects in plantation areas.
(DOC)

Appendix S3 List of landscape variables derived from GIS, the original source, and treatments to the data.
(DOC)

Appendix S4 Pearson's correlation coefficients between landscape variables at the grid level (17×17 km).
(DOC)

Appendix S5 Pearson's correlation coefficients between landscape variables at the transect level (extracted from 500 meter buffers surrounding the start- and end-points of each 1-km transect).
(DOC)

Appendix S6 Pearson's correlation coefficients for manual covariates.
(DOC)

Appendix S7 Pearson's correlation coefficients for manual plantation-specific covariates.
(DOC)
