# Supplementary material for: Tigers Need Cover: Multi-Scale Occupancy Study of the Big Cat in Sumatran Forest and Plantation Landscapes
Source: PLoS One. 2012 Jan 23;7(1):e30859. doi: 10.1371/journal.pone.0030859 (PMC3264627; doi:10.1371/journal.pone.0030859)
Supplement: Appendix S1 — List of habitat and environmental variables (manual covariates) collected in every 100-m segment along 1-km transects in forest and plantation landscapes of central Sumatra. (DOC) [file pone.0030859.s001.doc]

Appendix S1. List of habitat and environmental variables (manual covariates) collected in every 100-m segment along 1-km transects in forest and plantation landscapes of central Sumatra.

| **Variable Name** | **Description & scale of measurement** |
| --- | --- |
| **TERRAIN** |  |
| Alt | Altitude or elevation in meter based on reading from GPS’ barometric altimeter |
| Slope | The level of slope: 0=flat to 5=very steep |
| **VEGETATION COVER** | |
| habdom | General coverage condition of the vegetation (0=very open to 5=very dense/close) |
| canopy | Coverage of canopy (>30 cm diameter trees): 0 = 0%, 1 = 1-20 %, 2 = 21-40%, 3 = 41-60 %, 4 = 61-80 %, 5 = 81-100 % |
| subcanopy | Coverage of sub-canopy (sapling and trees<30cm): 0 = 0%, 1 = 1-20 %, 2 = 21-40%, 3 = 41-60 %, 4 = 61-80 %, 5 = 81-100 % |
| ground | Ground cover (seedlings and herbaceous plants): 0 = 0%, 1 = 1-20 %, 2 = 21-40%, 3 = 41-60 %, 4 = 61-80 %, 5 = 81-100 % |
| lccode | Ordinal coding of landcover types assigned based on general impression on its relationship/distance (e.g., geographically, historically) to forest: forest=1, acacia=2, oilpalm=3, rubber=4, mix agriculture=5, coconut=6 |
| **DISTURBANCE** |  |
| logging | The level of disturbance from logging activities: 0=none to 5=very high |
| encroach | The level of disturbance from encroachment activities: 0=none to 5=very high |
| fire | The level of disturbance from, or risk of fires: 0=none to 5=very high |
| settlement | The level of disturbance from settlement: 0=none to 5=very high |
| hunting | The level of disturbance from, or risk of hunting activities: 0=none to 5=very high |
