# Supplementary material for: Tigers Need Cover: Multi-Scale Occupancy Study of the Big Cat in Sumatran Forest and Plantation Landscapes
Source: PLoS One. 2012 Jan 23;7(1):e30859. doi: 10.1371/journal.pone.0030859 (PMC3264627; doi:10.1371/journal.pone.0030859)
Supplement: Appendix S2 — Additional environmental variables (manual plantation-specific covariates) collected in every 100-m segment along transects in plantation areas. (DOC) [file pone.0030859.s002.doc]

Appendix S2. Additional environmental variables (manual plantation-specific covariates) collected in every 100-m segment along transects in plantation areas.

| **Variable Name** | **Description** | **Scale of Measurement** | |
| --- | --- | --- | --- |
| Age | Age of the plantation | Scores of 0 to 5 |  |
| Height | Height of the plantation | meter |  |
| Husbandry | The intensity of plantation husbandry or maintenance | Scores of 0 to 5 |  |
| Otherplant | The density of plants other than the main commodities | Scores of 0 to 5 |  |
| Leaflitter | The coverage of leaf litter | Scores of 0 to 5 |  |
| Humanact | The intensity of human activities | Scores of 0 to 5 |  |
| Plantint | Space interval between plants of the main commodity species | Meter |  |
| Rotation | The rotation of plant since the area was opened from natural forest or other landcover types | Number |  |
