# Supplementary material for: Tigers Need Cover: Multi-Scale Occupancy Study of the Big Cat in Sumatran Forest and Plantation Landscapes
Source: PLoS One. 2012 Jan 23;7(1):e30859. doi: 10.1371/journal.pone.0030859 (PMC3264627; doi:10.1371/journal.pone.0030859)
Supplement: Appendix S3 — List of landscape variables derived from GIS, the original source, and treatments to the data. (DOC) [file pone.0030859.s003.doc]

Appendix S3. List of landscape variables derived from GIS, the original source, and treatments to the data.

| No | Variable | Original Source | Treatment & Range of Values |
| --- | --- | --- | --- |
| *1* | *Distance to major public road (“DtMPRd”)* | Road data from Indonesian National Mapping Agency (BAKOSURTANAL) | The original data were corrected for known errors. *Distance to road* raster data were derived using FindDistance in ArcView 3.2. Values range from 0 to 96,112 (x̄ = 15,641) meter. |
| *2* | *Distance to centroid forest area*  *(“dtf05cr”)* | Forest-non forest classification done by WWF (Setiabudi 2006) based on satellite images (spatial resolution ~30 m) acquired in 2005 | Forest blocks were defined as contiguous forest area equal to or greater than 50,000 ha. Centroids of forest blocks were determined using’Generate Polygon centroid point’ in Hawth's Analysis Tools Version 3.27 *(*[*www.spatialecology.com*](http://www.spatialecology.com/)*).* Distance to the Forest centroid raster data were derived using FindDistance in ArcView 3.2. Spatial Analyst. Values range from 7810 to 94032 (x̄ = 41642) meter. |
| *3* | *Distance to forest edge (“Dtfedge07”)* | Forest-non forest classification done by WWF (Setiabudi 2006) based on satellite images (spatial resolution ~30 m) acquired in 2007 | Using XTools version 6/1/2001 extension in ArcView 3.2, convert forest polygons to polylines (forest edge).  Distance to the Forest edge raster data were derived using FindDistance in ArcView 3.2. Spatial Analyst. Values range from 0 to 47,539 (x̄ = 7,024) meter. |
| *4* | *Distance to centroid of protected areas (“Dtpacr”)* | Protected area map was available from Indonesian Ministry of Forestry, maintained and updated by WWF Indonesia GIS team | Centroids of protected areas were determined using’Generate polygon centroid point’ in Hawth's Analysis Tools Version 3.27 ([www.spatialecology.com](http://www.spatialecology.com/)).Distance to the Forest centroid raster data were derived using FindDistance in ArcView 3.2. Spatial Analyst. Values range from 2236 to 81844 (x̄ = 31,035) meter. |
| *5* | *Precipitation (“Precip”)* | Bioclim interpolated precipitation version 1.4 Release 3. Spatial resolution of 30 arc-second (~ 1 square km), http://www.worldclim.org/ (Hijmans et al. 2005). | Total annual precipitation (using raster calculator in spatial analyst, sum of 12 months’ precipitation from the original data). Data for the study area were clipped using SpatialAnalyst>Extraction>Extract by Mask. Values range from 1989 to 2830 (x̄ = 2509) mm. |
| *6* | *Distance to freshwater (“Dtwater”)* | Rivers, canals, and lakes data as separate files from Digital Chart of the World Vector (line and area), downloaded through <http://www.diva-gis.org/gData> | Separate files were combined into one feature file in ArcGIS 9.3.1. and distance to the freshwater raster data were derived using FindDistance in ArcView 3.2. Values range from 0 to 11,677 (x̄ = 3,176) meter. |
| *7* | *Altitude (“AltDEM”)* | CIAT DEM SRTM *(*Shuttle Radar Topographic Mission) *version 4.*  [*http://srtm.csi.cgiar.org/index.asp*](http://srtm.csi.cgiar.org/index.asp)  CIAT: International Center for Tropical Agriculture. These data were originally derived from USGS/NASA SRTM data (Jarvis et al. 2004), resampled to 250 m resolution. | No treatment. Values range from 1 to 910 (x̄ = 139) meter. |
| *8* | *Slope (“Slope”)* | Derived from DEM SRTM version 4  250 m resolution [*http://srtm.csi.cgiar.org/index.asp*](http://srtm.csi.cgiar.org/index.asp),  (Jarvis et al. 2004) | Slopes (in percent) were derived from DEM SRTM in ArcGIS 9.3.1. using Spatial Analyst Tools. Values range from 0 to 22 (x̄ = 1.4) percent. |
| *9* | *Distance to Deforestation 06 to 07 (“Dtdef0607”)* | Forest-non forest classification available from a series of years: 1982, 1988,1996,2000, 2002, 2004, 2005, 2006, 2007 *(Uryu et al. 2007)*. | Deforestation or forest loss were calculated using forest cover layers from two different years (2006 and 2007, the available layers closest to the time of survey). Deforested areas were identified using Erase function in ET GeoWizard. Values range from 0 to 26,019 (x̄ = 7196) meter. |
| *10* | *Forest area 07 (“For07Area”)* | Forest-non forest classification(Uryu et al. 2007) | Forest area within each grid cell was calculated using *Hawth's Analysis Tools Version 3.27 (*[*www.spatialecology.com*](http://www.spatialecology.com/)*).* Values range from 0 to 288,090,761 (x̄ = 72,811,383) m^2^. |
