# Supplementary material for: Tigers Need Cover: Multi-Scale Occupancy Study of the Big Cat in Sumatran Forest and Plantation Landscapes
Source: PLoS One. 2012 Jan 23;7(1):e30859. doi: 10.1371/journal.pone.0030859 (PMC3264627; doi:10.1371/journal.pone.0030859)
Supplement: Appendix S4 — Pearson's correlation coefficients between landscape variables at the grid level (17×17 km). (DOC) [file pone.0030859.s004.doc]

Appendix S4. Pearson’s correlation coefficients between landscape variables at the grid level (17x17 km).

|  | Def0607 | For07Area | AltDEM | dtf05cr | dtpacr | Dtmprd | Precip |
| --- | --- | --- | --- | --- | --- | --- | --- |
| Def0607 | 1.00 |  |  |  |  |  |  |
| For07Area | 0.22 | 1.00 |  |  |  |  |  |
| AltDEM | 0.03 | 0.07 | 1.00 |  |  |  |  |
| dtf05cr | -0.23 | -0.56 | -0.07 | 1.00 |  |  |  |
| dtpacr | 0.04 | -0.37 | -0.02 | 0.61 | 1.00 |  |  |
| Dtmprd | 0.10 | 0.25 | -0.27 | -0.01 | 0.01 | 1.00 |  |
| Precip | -0.13 | -0.06 | 0.47 | 0.09 | 0.09 | -0.40 | 1.00 |

Note: Dtdef0607=Distance to deforested area 2006 to 2007, For07Area= Forest area 2007, AltDEM=altitude, dtf05cr= Distance to centroid forest area, Dtpacr= Distance to centroid of protected areas, Dtmprd=Distance to major public road, Precip=precipitation
