# Supplementary material for: Tigers Need Cover: Multi-Scale Occupancy Study of the Big Cat in Sumatran Forest and Plantation Landscapes
Source: PLoS One. 2012 Jan 23;7(1):e30859. doi: 10.1371/journal.pone.0030859 (PMC3264627; doi:10.1371/journal.pone.0030859)
Supplement: Appendix S5 — Pearson's correlation coefficients between landscape variables at the transect level (extracted from 500 meter buffers surrounding the start- and end-points of each 1-km transect). (DOC) [file pone.0030859.s005.doc]

Appendix S5. Pearson’s correlation coefficients between landscape variables at the transect level (extracted from 500 meter buffers surrounding the start- and end-points of each 1-km transect).

|  | dtmprd | precip | dtfedge07 | dtdef0607 | dtf05cr | dtpacr | dtwater | altDEM | slope |
| --- | --- | --- | --- | --- | --- | --- | --- | --- | --- |
| dtmprd | 1.00 |  |  |  |  |  |  |  |  |
| precip | -0.39 | 1.00 |  |  |  |  |  |  |  |
| dtfedge07 | -0.04 | -0.05 | 1.00 |  |  |  |  |  |  |
| dtdef0607 | 0.15 | -0.27 | **0.60** | 1.00 |  |  |  |  |  |
| dtf05cr | 0.08 | 0.08 | 0.37 | 0.26 | 1.00 |  |  |  |  |
| dtpacr | -0.11 | -0.03 | 0.24 | 0.23 | 0.27 | 1.00 |  |  |  |
| dtwater | -0.09 | -0.14 | 0.04 | 0.09 | 0.03 | 0.15 | 1.00 |  |  |
| altDEM | 0.01 | -0.25 | -0.09 | 0.21 | 0.00 | -0.26 | 0.03 | 1.00 |  |
| slope | 0.10 | -0.37 | -0.08 | 0.18 | -0.01 | -0.19 | 0.05 | **0.81** | 1.00 |

Note: Dtmprd=Distance to major public road, Precip=precipitation, Dtfedge07=distance to forest edge, Dtdef0607=Distance to deforested area 06 to 07, dtf05cr= Distance to centroid forest area, Dtpacr= Distance to centroid of protected areas, dtwater= distance to freshwater, AltDEM=altitude, slope=slope
