# Supplementary material for: Tigers Need Cover: Multi-Scale Occupancy Study of the Big Cat in Sumatran Forest and Plantation Landscapes
Source: PLoS One. 2012 Jan 23;7(1):e30859. doi: 10.1371/journal.pone.0030859 (PMC3264627; doi:10.1371/journal.pone.0030859)
Supplement: Appendix S6 — Pearson's correlation coefficients for manual covariates. (DOC) [file pone.0030859.s006.doc]

Appendix S6. Pearson’s correlation coefficients for manual covariates.

|  | overall | canopy | sub-canopy | under-story | logging | encro-achment | fire-risk | settle-ment | hunting | slope |
| --- | --- | --- | --- | --- | --- | --- | --- | --- | --- | --- |
| overall | 1.00 |  |  |  |  |  |  |  |  |  |
| canopy | 0.45 | 1.00 |  |  |  |  |  |  |  |  |
| subcanopy | 0.34 | 0.00 | 1.00 |  |  |  |  |  |  |  |
| understory | -0.07 | 0.08 | -0.04 | 1.00 |  |  |  |  |  |  |
| logging | -0.13 | -0.10 | -0.14 | -0.06 | 1.00 |  |  |  |  |  |
| encroachment | -0.38 | -0.31 | -0.28 | 0.01 | 0.31 | 1.00 |  |  |  |  |
| firerisk | -0.02 | -0.24 | -0.18 | -0.09 | 0.35 | 0.26 | 1.00 |  |  |  |
| settlement | -0.03 | -0.02 | -0.12 | 0.03 | -0.01 | 0.10 | 0.07 | 1.00 |  |  |
| hunting | 0.10 | -0.07 | -0.07 | -0.02 | 0.20 | 0.23 | 0.21 | 0.33 | 1.00 |  |
| slope | 0.07 | 0.30 | 0.17 | 0.11 | 0.03 | 0.01 | -0.16 | -0.13 | -0.10 | 1.00 |
| alt | 0.21 | 0.33 | 0.12 | -0.14 | 0.04 | -0.11 | -0.07 | -0.15 | -0.11 | 0.57 |
