# Supplementary material for: Tigers Need Cover: Multi-Scale Occupancy Study of the Big Cat in Sumatran Forest and Plantation Landscapes
Source: PLoS One. 2012 Jan 23;7(1):e30859. doi: 10.1371/journal.pone.0030859 (PMC3264627; doi:10.1371/journal.pone.0030859)
Supplement: Appendix S7 — Pearson's correlation coefficients for manual plantation-specific covariates. (DOC) [file pone.0030859.s007.doc]

Appendix S7. Pearson’s correlation coefficients for manual plantation-specific covariates.

|  | Age | Tree-Height | Hus-bandry | Other-Plants | Leaf-Litter | Human-Activities | Plan-Intervals |
| --- | --- | --- | --- | --- | --- | --- | --- |
| Age | 1 |  |  |  |  |  |  |
| TreeHeight | 0.54 | 1 |  |  |  |  |  |
| Husbandry | -0.26 | -0.09 | 1 |  |  |  |  |
| OtherPlants | 0.40 | 0.26 | -0.38 | 1 |  |  |  |
| LeafLitter | 0.59 | 0.29 | -0.12 | 0.12 | 1 |  |  |
| HumanActivities | -0.08 | 0.14 | -0.03 | -0.18 | -0.17 | 1 |  |
| PlanIntervals | 0.09 | -0.14 | 0.03 | 0.01 | 0.05 | -0.23 | 1 |
| Rotation | 0.14 | 0.22 | 0.29 | -0.35 | 0.33 | 0.28 | -0.29 |
